# Supplementary material for: PH-SHOWOA: Parallel hybrid SHO-WOA for VRPSPDTW
Source: PLoS One. 2026 Mar 27;21(3):e0343262. doi: 10.1371/journal.pone.0343262 (PMC13028424; doi:10.1371/journal.pone.0343262)
Supplement: Attachment — Submitted filename: PONE-D-25-56377_reviewer.docx [file pone.0343262.s001.docx]

Title should be more clear in title: "PH-SHOWOA: Parallel Hybrid SHO-WOA for VRPSPDTW”

Keywords should have: VRPSPDTW, Parallel Hybrid SHO-WOA

Abstract repeats several ideas (NP-hardness, exploration–exploitation, benchmark performance) and is very dense; trim general VRP motivation and allocate 1–2 sentences to specific numerical or qualitative improvements versus baselines.

The main novelty is in the solution method on VRPSPDTW (the PH‑SHOWOA hybrid and its mechanisms), then the most relevant and important part of the related work must focus on VRP/VRPSPDTW solution methods, not on application stories alone.

Also, summarize how SHO has been used and hybridized (e.g., with local search, opposition learning) and how WOA has been extended or hybridized. Explicitly note that no prior work has combined SHO and WOA for VRPSPDTW which positions your method as a new solution strategy in the VRP field.

**Missing citations**: The paper should cite:

- HSHO for FCCVRP (shows SHO works for VRP)
- hGWOA for CVRP (shows WOA works for VRP)
- SH-WOA for other applications (shows the combination works)

The problem definition section is seriously hindered by unclear structure, inconsistent notation, and several logical flaws that make the mathematical formulation difficult to follow and, in places, incorrect. The authors mix descriptive text, notation, equations, and commentary in a single continuous block, which prevents readers from clearly identifying the sets, parameters, variables, objective, and constraints that define the VRPSPDTW. Several definitions are ambiguous or incomplete for example, the statement that each node has “five attributes” is followed by a list of only four. Also, most formulas are not numbered.

A major presentation issue in the problem-definition section is that it contains several significant errors in presentation, consistency, and notation that need to be corrected. some constraints use informal notation (e.g., indicator functions, “1[·]”) without full definition, and the capacity/time-window constraints are described mostly in words rather than as explicit inequalities tied to the recursion equations for arrival/departure times and load.

Additionally, the section shifts between single-objective and multi-objective interpretations without clarifying which is adopted in the actual model. you mix the notation “VRPSPDTW” and “MO‑VRPSPDTW” without clearly distinguishing whether your computational work is single-objective or truly multi-objective; this creates confusion about the exact optimization problem being solved.

Your formulation is very similar to that used in VRPSPDTW algorithms such as MATE (memetic search) and VNS‑BSTS, which also adopt a lexicographic NV/TD cost function on WC instances:

Liu, S., Tang, K., & Yao, X. (2021). Memetic search for vehicle routing with simultaneous pickup-delivery and time windows. *Swarm and Evolutionary Computation*, *66*, 100927.

Overall, the problem definition requires substantial rewriting.

The description of the exploration–exploitation roles is inconsistent: in several places SHO is labeled “exploratory” and WOA “exploitative”, while the earlier text (and some algorithmic choices) suggest the opposite; this inconsistency makes it hard to understand the intended design logic. The prose around Algorithms 3–5 repeats similar sentences about “guided crossover, mutation, feasibility checks” multiple times, making the section verbose without adding insight.

It combines two existing metaheuristics (SHO and WOA) without substantial theoretical justification for why this specific combination is superior to other possible hybridizations. The claim of being the "first application of SHO and WOA to VRPSPDTW" is insufficient for a major contribution.

No testing on larger instances (>100 customers) despite claiming scalability

The paper addresses an important problem and shows promising results on some instances. However, it suffers from limited novelty, weak experimental validation, and presentation issues. The authors should address these questions:

Why specifically combine SHO and WOA rather than other metaheuristics?

How does performance scale with problem size beyond 100 customers?

Make it explicit that only 65 WC instances are used and clarify whether “newly derived large-scale instances” actually appear; if they do not appear in the tables, remove the phrase from abstract and contributions.

What is the actual parallel speedup achieved?

Why does the algorithm fail on RCdp instances compared to MA-FIRD?

Have you considered adaptive mechanisms for the pHybrid parameter?

This sentence is not complete: “*Declaration of AI and AI-assisted technologies in the writing process. During the preparation of this work, the author(s) used ChatGPT to improve and proofread the English of the paper. After using this tool/service, the author(s) reviewed and edited the”*

Complexity of algorithms is better to be clear.

The SHO and WOA parameters should be explained how these parameters were chosen or tuned, and whether any sensitivity analysis was done.

It is better to add statistical tests, convergence/sensitivity plots

(log TD vs iterations) and Boxplots (NV/TD distributions).

Regarding writing & technical, language: Generally clear but:

- Long sentences (avg 28 words)
- Repetition: "exploration-exploitation”.
- Typos: "publiation", "Parrallel", broken math
- Required: Professional proofreading.
