# Supplementary material for: PH-SHOWOA: Parallel hybrid SHO-WOA for VRPSPDTW
Source: PLoS One. 2026 Mar 27;21(3):e0343262. doi: 10.1371/journal.pone.0343262 (PMC13028424; doi:10.1371/journal.pone.0343262)
Supplement: Attachment — Submitted filename: Response to Reviewers_PONE-D-25-56377.docx [file pone.0343262.s003.docx]

Dear EiC and reviewers,

First of all, we would like to take this opportunity to thank you for your kind comments on the paper ***“A Parallel Hybrid Meta-heuristic Method for Solving Vehicle Routing Problems with Simultaneous Pickup and Delivery and Time Windows.”***

As per your suggestion, we have carefully revised the manuscript by taking into consideration all the comments. All changes are highlighted in the manuscript. The details of the revision are as follows:

**Response to EiC:**

1. Please ensure that your manuscript meets PLOS ONE's style requirements, including those for file naming. The PLOS ONE style templates can be found at

https://journals.plos.org/plosone/s/file?id=wjVg/PLOSOne_formatting_sample_main_body.pdf and https://journals.plos.org/plosone/s/file?id=ba62/PLOSOne_formatting_sample_title_authors_affiliations.pdf

**🡺 Response**: We have carefully revised the manuscript following the style requirements of PLOS ONE.

2. Please note that PLOS One has specific guidelines on code sharing for submissions in which author-generated code underpins the findings in the manuscript. In these cases, we expect all author-generated code to be made available without restrictions upon publication of the work. Please review our guidelines at https://journals.plos.org/plosone/s/materials-and-software-sharing#loc-sharing-code and ensure that your code is shared in a way that follows best practice and facilitates reproducibility and reuse.

**🡺 Response**: We have committed our code at the link: https://github.com/phuongtram/PH-SHOWOA

3. Thank you for stating the following in the Acknowledgments Section of your manuscript:

[The authors are grateful to the anonymous referees for carefully reading the manuscript and providing constructive comments, which helped substantially improve the paper. This work was supported by the project “Research and propose methods to solve transport optimization problems,” which received funding from the Information Technology Faculty (FIT), Nong Lam University - Thu Duc City - Ho Chi Minh City - Vietnam, with code project CS-CB23-CNTT-02.]

We note that you have provided funding information that is not currently declared in your Funding Statement. However, funding information should not appear in the Acknowledgments section or other areas of your manuscript. We will only publish funding information present in the Funding Statement section of the online submission form.

Please remove any funding-related text from the manuscript and let us know how you would like to update your Funding Statement. Currently, your Funding Statement reads as follows:

[The author(s) received no specific funding for this work.]

Please include your amended statements within your cover letter; we will change the online submission form on your behalf.

**🡺 Response**: We have removed funding from the manuscript. The full version of the funding statement is included at the end of the cover letter.

**Funding**

This work was supported by the project “Research and propose methods to solve transport optimization problems,” which received funding from the Information Technology Faculty (FIT), Nong Lam University - Ho Chi Minh City, Vietnam, with project code CS-CB23-CNTT-02.

4. Please note that your Data Availability Statement is currently missing the DOI/accession number of each dataset OR a direct link to access each database. If your manuscript is accepted for publication, you will be asked to provide these details on a very short timeline. We therefore suggest that you provide this information now, though we will not hold up the peer review process if you are unable.

**🡺 Response**: The datasets used for experiments are well-known benchmark instances and introduced in Section 5.1. Accordingly, datasets used from the following publication.

Wang, H. F., & Chen, Y. Y. (2012). A genetic algorithm for the simultaneous delivery and pickup problems with time window. Computers & industrial engineering, 62(1), 84-95. Datasets are available from https://github.com/senshineL/VRPenstein/tree/master

**Response to Reviewer #1:**

The manuscript presents an interesting and potentially useful hybrid metaheuristic (PH‑SHOWOA) for the VRPSPDTW and reports extensive experiments on benchmark instances. The overall structure is clear and the topic fits well within the scope of the journal. However, the paper currently falls short of publication standards and requires substantial revision before it can be considered further. The main concerns relate to (i) clarity and internal consistency of the problem and algorithmic description, (ii) fairness and completeness of the computational comparisons, and (iii) the strength of the performance and novelty claims. The attached files mentioned the comments. I encourage the authors to carefully clean and streamline the algorithm descriptions, provide a fully reproducible parameter specification, and, where possible, run competing methods under common experimental conditions or temper the strength of the comparative statements. Adding formal statistical analyses would also greatly strengthen the empirical section. With these issues addressed and the claims aligned more closely with the evidence, the work could make a valuable contribution to the literature on VRPSPDTW solution methods.

1) **Title should be more clear in title: "PH-SHOWOA: Parallel Hybrid SHO-WOA for VRPSPDTW”**

**🡺 Response**: We have revised the title of the manuscript as follows: PH-SHOWOA: Parallel Hybrid SHO-WOA for VRPSPDTW

2) **Keywords** should have: VRPSPDTW, Parallel Hybrid SHO-WOA

**🡺 Response**: We have added keywords as the reviewer’s suggestion.

Keywords. VRPSPDTW, Parallel Hybrid SHO-WOA, meta-heuristic algorithm, Combinatorial optimization, Industrial application.

3) **Abstract** repeats several ideas (NP-hardness, exploration–exploitation, benchmark performance) and is very dense; trim general VRP motivation and allocate 1–2 sentences to specific numerical or qualitative improvements versus baselines.

**🡺 Response**: We have carefully revised the Abstract section to remove repeated ideas and trim general VRP motivation and a brief introduction to the experimental results in comparison to selected baselines.

4) The main novelty is in the solution method on VRPSPDTW (the PH‑SHOWOA hybrid and its mechanisms), then the most relevant and important part of the related work must focus on VRP/VRPSPDTW solution methods, not on application stories alone.

**🡺 Response**: We have carefully revised the related works to mainly focus on the approaches to VRP/VRPSPDTW. We have added 3 paragraphs for these approaches in Related works section. The changes are highlighted in the manuscript.

5) Also, summarize how SHO has been used and hybridized (e.g., with local search, opposition learning) and how WOA has been extended or hybridized. Explicitly note that no prior work has combined SHO and WOA for VRPSPDTW which positions your method as a new solution strategy in the VRP field.

**🡺 Response**: We have added text on Subsection 4.1. to clarify how SHO and WOA are combined with other algorithms.

6) **Missing citations**: The paper should cite:

- HSHO for FCCVRP (shows SHO works for VRP)
- hGWOA for CVRP (shows WOA works for VRP)
- SH-WOA for other applications (shows the combination works)

**🡺 Response**: We have cited the following references in Related Works.

- Pham, V. H. S., Nguyen, V. N., & Dang, N. T. N. (2025). Novel hybrid swarm intelligence algorithm for solving the capacitated vehicle routing problem efficiently. Evolutionary Intelligence, 18(3), 61.
- Utama, D. M., Yurifah, A., & Garside, A. K. (2023). A novel hybrid spotted hyena optimizer: An algorithm for fuel consumption capacitated vehicle routing problem. International Journal of Technology, 14(5), 1049-1059.
- Abidi, M. H., Umer, U., Mohammed, M. K., Aboudaif, M. K., & Alkhalefah, H. (2020). Automated maintenance data classification using recurrent neural network: Enhancement by spotted hyena-based whale optimization. Mathematics, 8(11), 2008.

7) The problem definition section is seriously hindered by unclear structure, inconsistent notation, and several logical flaws that make the mathematical formulation difficult to follow and, in places, incorrect. The authors mix descriptive text, notation, equations, and commentary in a single continuous block, which prevents readers from clearly identifying the sets, parameters, variables, objective, and constraints that define the VRPSPDTW. Several definitions are ambiguous or incomplete for example, the statement that each node has “five attributes” is followed by a list of only four. Also, most formulas are not numbered.

**🡺 Response**: We have carefully revised the problem definition section. All definitions have been revised, and all equations have been reformatted in display mode to enhance clarity and readability.

8) A major presentation issue in the problem-definition section is that it contains several significant errors in presentation, consistency, and notation that need to be corrected. some constraints use informal notation (e.g., indicator functions, “1[·]”) without full definition, and the capacity/time-window constraints are described mostly in words rather than as explicit inequalities tied to the recursion equations for arrival/departure times and load.

**🡺 Response**: We have carefully revised the Problem Definition section in a more detailed way. The changes are highlighted in the manuscript.

9) Additionally, the section shifts between single-objective and multi-objective interpretations without clarifying which is adopted in the actual model. you mix the notation “VRPSPDTW” and “MO‑VRPSPDTW” without clearly distinguishing whether your computational work is single-objective or truly multi-objective; this creates confusion about the exact optimization problem being solved.

**🡺 Response**: We have revised the research problem as a single-objective problem as follows:

Among these, the Vehicle Routing Problem with Simultaneous Pickup and Delivery and Time Windows (VRPSPDTW) is particularly challenging, as it simultaneously considers capacity constraints, bidirectional flows, and strict service time windows. The integration of these constraints significantly enlarges the search space and increases the complexity of the solution.

10) Your formulation is very similar to that used in VRPSPDTW algorithms such as MATE (memetic search) and VNS‑BSTS, which also adopt a lexicographic NV/TD cost function on WC instances:

Liu, S., Tang, K., & Yao, X. (2021). Memetic search for vehicle routing with simultaneous pickup-delivery and time windows. *Swarm and Evolutionary Computation*, *66*, 100927.

**🡺 Response**: We have cited the reference that the reviewer mentioned.

11) Overall, the problem definition requires substantial rewriting.

**🡺 Response**: We have carefully revised the problem definition for clarity and ease of reading.

12) The description of the exploration–exploitation roles is inconsistent: in several places SHO is labeled “exploratory” and WOA “exploitative”, while the earlier text (and some algorithmic choices) suggest the opposite; this inconsistency makes it hard to understand the intended design logic. The prose around Algorithms 3–5 repeats similar sentences about “guided crossover, mutation, feasibility checks” multiple times, making the section verbose without adding insight.

**🡺 Response**: We have carefully revised the description of the exploration–exploitation roles that were used in the manuscript. Also, the prose around Algorithms 3 – 5 has been revised to reduce the repetition of these terms (“guided crossover, mutation, feasibility checks”).

13) It combines two existing metaheuristics (SHO and WOA) without substantial theoretical justification for why this specific combination is superior to other possible hybridizations. The claim of being the "first application of SHO and WOA to VRPSPDTW" is insufficient for a major contribution.

**🡺 Response**: We have carefully revised the manuscript Balance of Exploration and Exploitation to clarify the superiority of the hybridizations. Also, the claim “first application of SHO and WOA to VRPSPDTW” has been removed.

14) No testing on larger instances (>100 customers) despite claiming scalability

**🡺 Response**: Actually, our experiments were carried out on small- and medium-scale instances (up to 100 customer instances). Thus, we have revised the manuscript to clearly state the considered instance sizes and to align the discussion with these experimental settings.

15) The paper addresses an important problem and shows promising results on some instances. However, it suffers from limited novelty, weak experimental validation, and presentation issues. The authors should address these questions:

Why specifically combine SHO and WOA rather than other metaheuristics?

**🡺 Response**: We revised the abstract and provided a short explanation for the reason of combining SHO and WOA. The changes are highlighted in the Abstract.

The proposed method leverages the strength of both algorithms: SHO primarily supports population-level diversification, while WOA focuses on best-guided intensification.

16) How does performance scale with problem size beyond 100 customers?

**🡺 Response**: Our experiments were carried out on small- and medium-scale instances (up to 100 customer instances). Therefore, in the future, we intend to conduct experiments with larger customer instances.

17) Make it explicit that only 65 WC instances are used and clarify whether “newly derived large-scale instances” actually appear; if they do not appear in the tables, remove the phrase from abstract and contributions.

**🡺 Response**: We have carefully revised the abstract and the contributions. Since in the manuscript we focused on small- and large-scale instances(up to 100 customer instances).

18) What is the actual parallel speedup achieved?

**🡺 Response**: In subsection 4.12, we have provided more details to explain the prominent role of the proposed method, including the parallel mechanism.

19) Why does the algorithm fail on RCdp instances compared to MA-FIRD?

**🡺 Response**: We added subsection “6.1.	Robust improvements in routing efficiency” to explain the competition of the proposed algorithm in comparison to MA-FIRD on RCdp .

20) Have you considered adaptive mechanisms for the pHybrid parameter?

**🡺 Response**: In Subsection 4.1, we already incorporate an adaptive mechanism for the hybrid control parameter $p_{\mathrm{Hybrid}}$in **Algorithm 1 (Lines 7–9)**. Rather than being fixed, $p_{\mathrm{Hybrid}}$is dynamically adjusted as a function of the iteration index, gradually decreasing from SHO-dominated exploration in the early search phase to WOA-driven exploitation in later iterations. This time-varying strategy enables the algorithm to emphasize diversification at the beginning and intensification as convergence progresses, which is consistent with the classical exploration–exploitation trade-off in swarm intelligence algorithms.

21) This sentence is not complete: “*Declaration of AI and AI-assisted technologies in the writing process. During the preparation of this work, the author(s) used ChatGPT to improve and proofread the English of the paper. After using this tool/service, the author(s) reviewed and edited the paper”*

**🡺 Response**: Thank you very much. We have carefully revised the sentence.

22) Complexity of algorithms is better to be clear.

**🡺 Response**: We thank the reviewer for this helpful comment. In the revised manuscript, we have clarified the computational complexity of the proposed PH-SHOWOA in a dedicated subsection 4.11, with explicit variable definitions and direct references to Algorithm 1. Specifically, we analyze both the per-iteration and overall time complexity, showing that each individual update has worst-case complexity $\mathcal{O(}N)$, which results in an overall complexity of $\mathcal{O(}I\cdot P\cdot N)$, where $N$denotes the number of customers, $P$the population size, and $I$the number of iterations. We also discuss the effect of periodic local search and parallel execution on runtime and scalability. These clarifications improve the transparency and reproducibility of the proposed method.

23) The SHO and WOA parameters should be explained how these parameters were chosen or tuned, and whether any sensitivity analysis was done.

**🡺 Response**: We thank the reviewer for this insightful comment. In the revised manuscript, we have clarified the selection and tuning of the SHO and WOA parameters in the experimental settings section. Specifically, all algorithmic parameters follow the recommendations of the original SHO and WOA studies to ensure fairness and reproducibility. The exploration–exploitation control parameters ($h$ in SHO and $a$in WOA) are linearly decreased according to standard practice, while the stochastic coefficients are uniformly sampled at each iteration to maintain population diversity. For the proposed PH-SHOWOA, the hybrid control parameter is not fixed but adaptively adjusted over iterations (Algorithm 1, Lines 7–9), progressively shifting the search from SHO-based exploration to WOA-based exploitation. In addition, a limited sensitivity analysis was conducted on representative small and medium WC instances, indicating that the algorithm is robust to moderate parameter variations. Based on these observations, a single unified parameter setting was adopted for all experiments.

24) It is better to add statistical tests, convergence/sensitivity plots (log TD vs iterations) and Boxplots (NV/TD distributions).

**🡺 Response**: We have carefully revised the manuscript and added a subsection 5.2.3.2 Statistical test on 56 benchmark instances (Rdp, Cdp, and Rcdp).

25) Regarding writing & technical, language: Generally clear but:

- Long sentences (avg 28 words)
- Repetition: "exploration-exploitation”.
- Typos: "publiation", "Parrallel", broken math
- Required: Professional proofreading.

**Response**: We have carefully revised the English of the manuscript.

**Response to Reviewer #2:**

The paper makes a solid contribution to the VRPSPDTW literature by being the first to apply a hybrid SHO-WOA approach to this problem variant. The methodology is generally sound, combining population-based search with adaptive probability control, simulated annealing acceptance, periodic local search, and parallelization. The experimental evaluation is comprehensive, covering multiple instance categories and comparing against recent competitive algorithms. The paper is reasonably well-written with clear algorithm descriptions that support reproducibility. However, several areas require improvement to strengthen the contribution and meet publication standards. I recommend minor revision to address statistical validation, clarify algorithmic details, and enhance the performance analysis.

The paper has several notable strengths that support its contribution to the field. First, it addresses an important and challenging NP-hard problem with significant practical applications in modern logistics and sustainable transportation. The VRPSPDTW combines multiple real-world constraints, simultaneous pickup and delivery operations with strict time windows, making it highly relevant for contemporary distribution systems.

Second, the methodological novelty of applying SHO and WOA to VRPSPDTW is genuine and meaningful. While these bio-inspired algorithms have been successfully applied to various optimization problems, their combination for this specific routing variant represents new territory. The hybrid framework intelligently leverages SHO's exploitative hunting strategy alongside WOA's exploratory bubble-net behavior, creating a complementary search mechanism.

Third, the experimental evaluation is comprehensive and rigorous in scope. Testing on 65 benchmark instances across different customer distribution patterns (random, clustered, and mixed) provides thorough coverage of problem characteristics. The comparison against three recent state-of-the-art methods (Co-GA from 2012, ACO-DR from 2023, and MA-FIRD from 2024) situates the work appropriately within current research.

Fourth, the results demonstrate clear practical value, particularly for clustered customer scenarios. The algorithm achieves best performance on 14 of 18 Cdp instances for both number of vehicles and total distance, with some solutions showing distance reductions exceeding 30% compared to competitors. Even on more challenging instance types, the method frequently produces competitive or superior total distances, which directly translates to operational cost savings.

Finally, the paper provides detailed algorithm descriptions through eight well-structured pseudocode blocks. This level of detail, including the initialization procedure, hybrid update mechanisms, crossover and mutation operators, and combined local search, supports reproducibility and enables other researchers to build upon this work.

Required Revisions:

1) **Statistical Validation**

The paper currently presents only mean values across 30 independent runs without any statistical significance testing. While the results appear promising, readers cannot determine whether the observed improvements represent genuine algorithmic advantages or merely random variation. This is particularly important given the stochastic nature of metaheuristic algorithms and the relatively small performance differences in some instances.

The authors should add appropriate statistical tests to validate their claims. A Wilcoxon signed-rank test would be suitable for pairwise comparisons between PH-SHOWOA and each baseline algorithm (SHO, WOA, and ideally the competing methods). For comparisons across multiple instance categories, a Friedman test followed by post-hoc analysis would provide more robust validation. Additionally, including standard deviations or confidence intervals in Tables 4-8, or providing box plots for representative instances, would help readers assess solution variability and algorithm stability. A summary table presenting aggregate statistical results across instance categories would strengthen the experimental section considerably.

**🡺 Response**: We have applied a solid statistical test on 56 benchmark instances (Rdp, Cdp, and Rcdp) (Subsection 5.2.3.2).

2) **Algorithm Clarity and Completeness**

Several algorithmic details remain unclear or undefined, which hinders complete understanding and reproducibility. Algorithm 4 (BasedOnWOAUpdate) references "ApplyRandomMultiRouteOperation" at line 24, but this operation is never formally defined. Similarly, "ApplyRandomOperation" appears in multiple algorithms without specification. While these may be standard VRP neighborhood operators, explicit definitions or references are necessary for clarity.

**🡺 Response**: We have carefully revised the manuscript, and the explanation of these mechanisms is provided in subsection 4.3. (Strategy for a Parallel Hybrid Based on SHO–WOA Framework).

The feasibility checking and repair mechanisms are mentioned throughout the paper but never formally specified. The phrase "if infeasible then repair" appears frequently, but readers need to understand exactly how capacity violations and time window conflicts are detected and corrected. I recommend adding a dedicated algorithm (perhaps Algorithm 9) that formalizes the feasibility checking and repair procedures, or at minimum, providing a clear textual description in Section 4.

**🡺 Response**: Thank you so much for the suggestion. We have added algorithms 9 and 10 for such tasks.

3) **Presentation and Writing Quality**

While the paper is generally well-written, several presentation issues should be addressed. The notation inconsistency throughout the manuscript is distracting. Section 3 introduces "MO-VRPSPDTW" (multi-objective VRPSPDTW) but this term is barely used afterward, with most of the paper referring simply to "VRPSPDTW." Either use the MO prefix consistently if discussing multi-objective optimization, or remove it and focus on the weighted-sum hierarchical objective. Similarly, subscript notation varies between h_{i,j} and h_i in different sections, standardize throughout.

**🡺 Response**: We have revised all notions to ensure their consistency.

4) Tables 6-8 mention color coding in the text (gray and yellow shading) to highlight different performance levels, but these colors are not visible in the submitted manuscript. Either ensure the colors appear in the final PDF or remove references to them and rely solely on textual indicators like bold and ** notation.

**🡺 Response**: We have revised the tables and used bold and ** notation to highlight different performance levels.

5) Section 4.9 (Discussion of the proposed method) largely repeats content from the introduction regarding contributions and novelty. This section could be streamlined to avoid redundancy, perhaps by focusing more on the technical rationale for design decisions rather than restating high-level contributions. The conclusion (Section 7) is relatively brief given the paper's length and could be expanded to better synthesize findings, acknowledge limitations more explicitly, and provide more specific future research directions.

**🡺 Response**: We have streamlined Section 4.9 and provided more details of the techniques used in algorithms.

=============

Thank you very much again for your valuable comments.

Best regards,

Van Du Nguyen
